# Tetrahydropyrazolo[1,5-*a*]Pyrimidine-3-Carboxamide and *N*-Benzyl-6′,7′-Dihydrospiro[Piperidine-4,4′-Thieno[3,2-*c*]Pyran] Analogues with Bactericidal Efficacy against *Mycobacterium tuberculosis* Targeting MmpL3

**DOI:** 10.1371/journal.pone.0060933

**Published:** 2013-04-17

**Authors:** Modesto J. Remuiñán, Esther Pérez-Herrán, Joaquín Rullás, Carlos Alemparte, María Martínez-Hoyos, David J. Dow, Johnson Afari, Nalini Mehta, Jorge Esquivias, Elena Jiménez, Fátima Ortega-Muro, María Teresa Fraile-Gabaldón, Vickey L. Spivey, Nicholas J. Loman, Mark J. Pallen, Chrystala Constantinidou, Douglas J. Minick, Mónica Cacho, María José Rebollo-López, Carolina González, Verónica Sousa, Iñigo Angulo-Barturen, Alfonso Mendoza-Losana, David Barros, Gurdyal S. Besra, Lluís Ballell, Nicholas Cammack

**Affiliations:** 1 Diseases of the Developing World, GlaxoSmithKline, Tres Cantos, Madrid, Spain; 2 Molecular and Cellular Technologies, GlaxoSmithKline, Stevenage, United Kingdom; 3 School of Biosciences, University of Birmingham, Edgbaston, United Kingdom; 4 Department of Analytical Chemistry, GlaxoSmithKline, Research Triangle Park, North Carolina, United States of America; University of Delhi, India

## Abstract

*Mycobacterium tuberculosis* is a major human pathogen and the causative agent for the pulmonary disease, tuberculosis (TB). Current treatment programs to combat TB are under threat due to the emergence of multi-drug and extensively-drug resistant TB. As part of our efforts towards the discovery of new anti-tubercular leads, a number of potent tetrahydropyrazolo[1,5-a]pyrimidine-3-carboxamide (THPP) and *N*-benzyl-6′,7′-dihydrospiro[piperidine-4,4′-thieno[3,2-c]pyran] (Spiro) analogues were recently identified against *Mycobacterium tuberculosis* and *Mycobacterium bovis BCG* through a high-throughput whole-cell screening campaign. Herein, we describe the attractive *in vitro* and *in vivo* anti-tubercular profiles of both lead series. The generation of *M. tuberculosis* spontaneous mutants and subsequent whole genome sequencing of several resistant mutants identified single mutations in the essential *mmpL3* gene. This ‘genetic phenotype’ was further confirmed by a ‘chemical phenotype’, whereby *M. bovis* BCG treated with both the THPP and Spiro series resulted in the accumulation of trehalose monomycolate. *In vivo* efficacy evaluation of two optimized THPP and Spiro leads showed how the compounds were able to reduce >2 logs bacterial cfu counts in the lungs of infected mice.

## Introduction

Despite the existence of treatments for Tuberculosis (TB), nine million people are currently infected and one and a half million die, each year [1],[2]. The disease also represents an escalating threat for global health, with the increasing prevalence of Multi Drug Resistant (MDR) and Extensively Drug Resistant (XDR) TB strains [3]. While the misuse of current anti-tuberculars is one of the contributing driving forces behind this trend, this situation is also a consequence of the nature of the treatment: a combination of at least three different drugs with known side-effects that must be taken for 6 months or longer. This situation often leads to poor patient compliance with a consequent rise in drug resistant strains and infection relapse cases. The WHO sponsored implementation of Directly Observed Treatment Short course [DOTS] [1] in which treatment compliance is monitored by healthcare workers has been successful when appropriately implemented (cure rate >90%). Despite this, the development of new drugs with novel modes of action (MoA) for the treatment of TB would likely still be the most cost-effective way of tackling the pandemic. Specifically, any new drug should be able to shorten the duration of treatment, avoid significant drug-drug interaction with current regimens, treat MDR as well as XDR-TB patients and be competitive in terms of cost with current drugs [4].

A quick historical analysis of the most commonly applied drug discovery strategies shows how ‘target based’ approaches have lately dominated drug discovery with the antibacterial and antitubercular fields being no exception. These bottom-up approaches have had limited success considering the amount of investment and the number of late stage clinical assets generated [5]. In the case of TB drug discovery these attrition rates become even more alarming given the limited number of validated targets that have gone on to be exploited for drug discovery purposes. Consequently, many researchers are turning towards phenotypic screens, where promising compounds are identified in antibacterial whole cell assays. As examples of this approach, a number of molecules currently exist at different stages of late pre-clinical and clinical development, the most advanced of them being SQ-109 [6], PA-824 [7] and Bedaquiline [8]. In addition to these, a number of promising leads [9],[10],[11] and candidates [12] further validate this shift from target-based programs to phenotypic screens. The often attractive anti-tubercular properties of these molecules have also spurred interest in mode of action (MoA) studies, leading to the postulation of new hypotheses for benzothiazinones [12], bedaquiline [8], PA-824 [13], SQ-109 [14], BM212 [15], adamantyl ureas [16] and benzimidazole C215 [17]. This, in time, could lead to new target-based screening efforts that will hopefully not be affected by the historically high attrition rates in antibacterial drug discovery.

As part of our continuous efforts towards the discovery of new anti-tubercular leads, a number of potent tetrahydropyrazolo[1,5-*a*]pyrimidine-3-carboxamide (THPP) and *N*-benzyl-6′,7′-dihydrospiro[piperidine-4,4′-thieno[3,2-c]pyran] (Spiro) analogues were recently identified against *Mycobacterium tuberculosis* and *Mycobacterium bovis* BCG [11]. Herein, we describe the attractive *in vitro* and *in vivo* pharmacological profiles of both lead series and demonstrate how these molecules interfere with trehalose dimycolate (TDM) production through mutations in the essential *mmpL*3 gene.

## Results

### Identification and description of THPP's and Spiros as anti-TB agents

**Figure 1:**
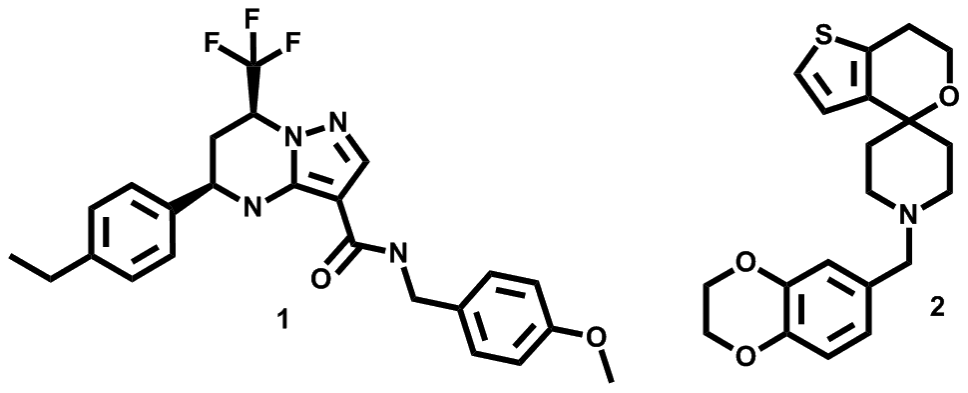
Chemical structures of 1 and 2. Absolute configuration of 1 was determined by circular dichroism (Protocol S3). Racemic mixture was separated by high performance liquid chromatography.

As a result of GSK's continued anti-mycobacterial phenotypic screening efforts, two new chemical scaffolds, namely tetrahydropyrazolo[1,5-*a*]pyrimidine-3-carboxamides (THPP's) and *N*-benzyl-6′,7′-dihydrospiro[piperidine-4,4′-thieno[3,2-*c*]pyrans] (Spiro's), were identified against *M. bovis BCG* (Figure 1) [11]. While in the case of THPP's, closely related analogues have been previously described in phenotypic screenings [18], the anti-tubercular properties of the novel Spiro hit have not been previously reported. A preliminary profiling of both hit structures (Table 1) showed how the compounds were endowed with potent and selective anti-tubercular activity. Compound **1** did not show initial signs of cytotoxicity at the maximum concentration tested where this compound was observed to be in solution. On the other hand, the Tox50 value determined for more soluble Spiro **2** was 36 µM. Interestingly, both representative molecules showed noticeable intracellular *in vitro* anti-tubercular activity against infected murine macrophages in addition to constant MIC values when assayed against an extensive panel of *M. tuberculosis* clinical isolates (MIC90). As primary issues, THPP **1** showed very low solubility in different media and Spiro **2** offered poor intrinsic clearance values in both mice and human microsomes.

**Table 1:** *In vitro* profile of compounds 1 and 2.

Compound	1	2
H37Rv MIC (µM)	0.3	0.3
*M. tuberculosis* MIC90 (µM) (108 strains)	0.6	0.6
Intracell. H37Rv MIC80 (µM)	0.16	0.63
Antibacterial panel IC50 (µM)	≥32	≥16
HepG2 Tox50 (µM)	>25*	36
ClogP	3.65	2.99
Cli (ml/min*g) mouse	0.69	>30
Cli (ml/min*g) human	0.29	25
Solubility CLND (µM)	5	266

* limited by solubility.

### *In vitro* TB profile – killing curves, MDR/XDR, frequency of resistant mutants

**Figure 2:**
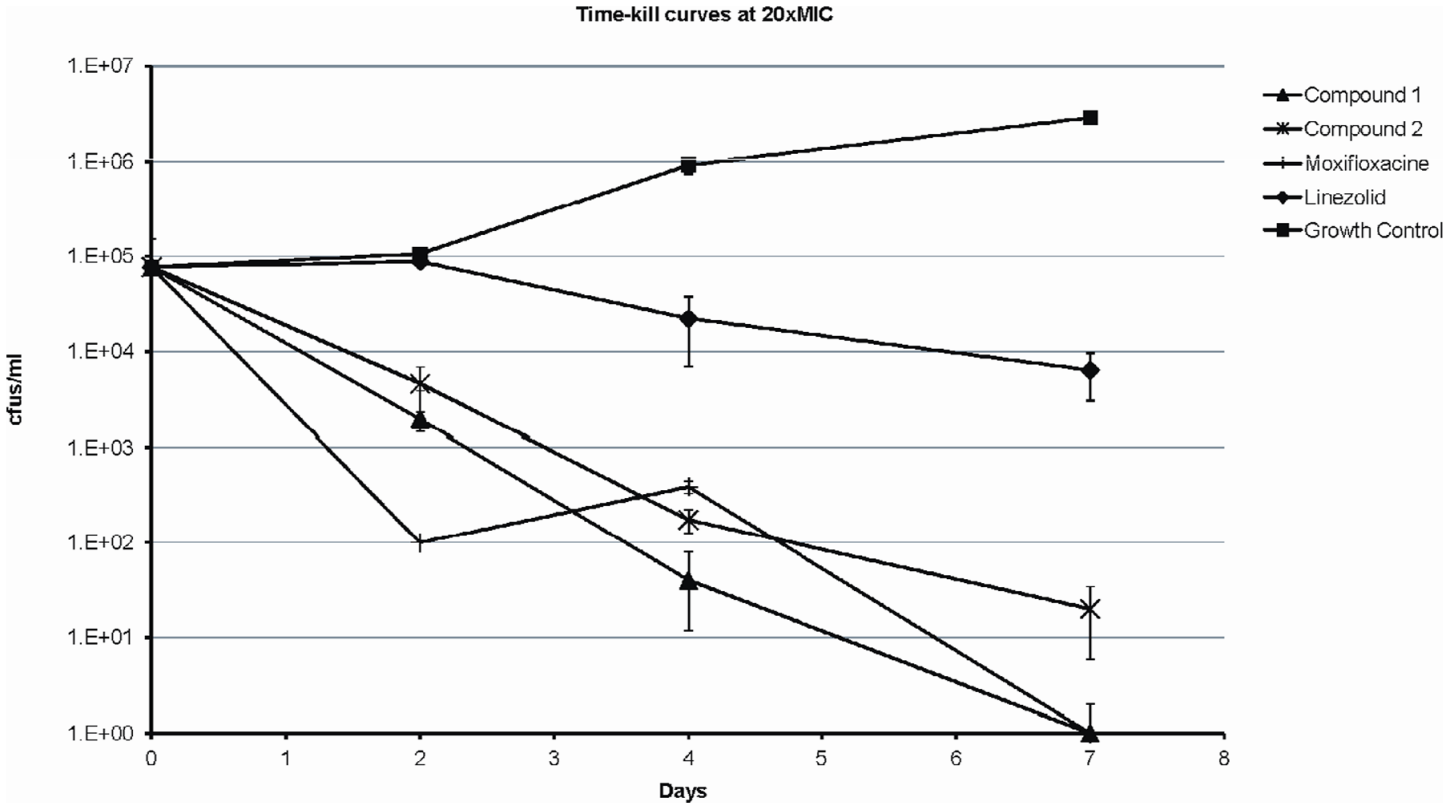
*M. tuberculosis* killing kinetics of 1 and 2 at 20x MIC. The number of cfus were quantified after incubation with the different compounds at different times in 10 ml of 7H9 10%ADC 0,05% Tween 80 medium containing 5 µM of 1, 7.5 µM of 2, 9.4 µg/ml of Linezolid, 1.2 µg/ml of Moxifloxacine and also for internal growth control. The mean and the standard deviations of at triplicate cultures of each point are shown.

The bactericidal potential of both **1** and **2** were assessed in killing rate experiments with Linezolid and Moxifloxacin as bacteriostatic and bactericidal controls, respectively (Figure 2). Bactericidal activity against *M. tuberculosis* was defined as a 99.9 % reduction of the initial inoculum after seven days of incubation with the compound. The results show that both the THPP and Spiro hits were able to reduce >3 log cfu counts after one week of treatment.

The frequency of spontaneous resistant mutant generation was also assessed. In both instances the frequency was within the same order of magnitude, being 3.6×10^−8^ mutant/cfus for compound **2** and 6.4×10^−8^ mutant/cfus for compound **1**. Both compounds were tested against *M. tuberculosis* H37Rv in the presence of serum and no significant shift was observed in the MIC values (data not shown). No major shifts in the *in vitro* MIC values were either observed against a panel of 125 different clinical isolates, including MDR and XDR strains (Table 2).

**Table 2:** Activity of 1 and 2 in clinical *M. tuberculosis* isolates.

		Range of MIC's (µM)		
Phenotype		Compound 1	Compound 2	Number of strains
Sensitive		0.16–0.6	0.3–1.25	60
	H	0.16–0.6	0.3–10	20
monoR	Rf	0.16–0.3	0.6–10	3
	Sm	0.16–0.6	0.3–10	11
MDR		0.16–2.5	0.3–1.25	19
XDR		0.3–5	0.3–1.25	12

H: Isoniazid; Rf: Rifampicin; Sm: Streptomycin.

### Identification of MmpL3 as a target of THPP 1 and Spiro 2

The elucidation of the cellular target is an important step towards the validation of the inhibitor family as a suitable therapeutic anti-tubercular agent. **1** and **2** exhibited encouraging MIC values against *M. tuberculosis* (Table 1) and thus were selected for use in strategies towards the identification of their target. Using these values, *M. tuberculosis* spontaneous resistant mutants were isolated at 10× MIC of each compound (Protocol S2). A total of 67 mutants were studied, 33 in respect of **1** and 34 for **2**. The resistant mutants contained one or more mutations allowing them to survive in the presence of the inhibitor, by either affecting drug accumulation within the cell or by preventing the inhibitory impact of the drug on the target itself. Thus identification of the mutation locus within a gene could indicate the drug target. Whole-genome sequencing analysis of 6 mutants (3 from each class) detected a single nucleotide polymorphism (SNP) compared to the *M. tuberculosis* H37Rv wild type sequence. In all cases, this SNP was found in *mmpL3* conferring a predicted amino acid alteration in the protein sequence. For **1** we observed A249P and A677V, and for **2** we found F255L and Y252C. For the remaining 61 mutants, *mmpL3* was specifically sequenced and 6 new SNPs were identified (Table 3). According to TraSH analysis, *mmpL3* is an essential gene [19]. As first attempt to confirm MmpL3 as the target of the two compounds and to establish cross-resistance, resistant mutants were tested against both chemical series (Table 4). The results clearly demonstrate that each *M. tuberculosis* resistant strain with a specific mutation raised against its respective inhibitor **1** and **2** was confirmed to be resistant. Despite that all mutations were located in *mmpL3*, no clear cross-resistance pattern was observed between THPP and SPIRO, compounds **1** and **2** (Table 4). Finally, it is also noteworthy all isolated mutants were found susceptible to classic antitubercular drugs (Moxifloxacin, Ethambutol, Linezolid).

**Table 3:** Genotypic characterization of spontaneous *M. tuberculosis* mutants isolated with 1 or 2.

Resistant mutants to Compound 1		
n = 33	Mutation	AA change
1 Mutant	g2137a	Val713Met
27 mutants	g745c	Ala249Pro
2 mutants	t1931g	Phe644Cys
3 mutants	c2030t	Ala677Val

Resistant mutants to Compound 2		
n = 34	Mutation	AA change
7 Mutants	a755g	Tyr252Cys
7 mutants	t763c	Phe255Leu
4 mutants	c765a	Phe255Leu
9 mutants	t763g	Phe255Val
1 mutants	t875g	Ile292Ser
2 mutants	t875c	Ile292Thr
4 mutants	g1772t	Ser591Ile

**Table 4:** Cross-Resistance study between 1 and 2.

	Ratio MICmut/MICwt									
	H37Rv mutants selected with Compound 1				H37Rv mutants selected with Compound 2					
MmpL3 Mutations	Ala249Pro	Ala677Val	Phe644Cys	Val713Met	Phe255Val	Ile292Thr	Ile292Ser	Tyr252Cys	Phe255Leu	Ser591Ile
**Compound 1**	>8	>8	>8	>70	2.0	0.5	1.0	4.0	8.0	>8
**Compound 2**	1.1	1.6	8.3	125.0	33.3	33.3	≥33.3	≥33.3	≥33.3	≥33.3
**Compound 3**	8.0	11.3	22.5	ND	1.9	0.7	0.7	3.8	15.0	30.0
**Compound 4**	0.4	0.3	16.0	ND	16.0	2.0	12.0	>16	>16	>16
**Moxifloxacin**	1.0	1.0	1.0	1.0	1.0	1.0	1.0	1.0	0.5	1.0
**Ethambutol**	0.5	1.0	1.0	ND	1.0	1.0	1.0	0.8	1.0	1.0
**Linezolid**	0.5	1.0	1.0	1.0	1.0	1.0	1.0	1.0	1.0	1.0

### 1 and 2 treatments give rise to mutations in MmpL3 and lead to an accumulation of trehalose monomycolate (TMM) in mycobacteria

**Figure 3:**
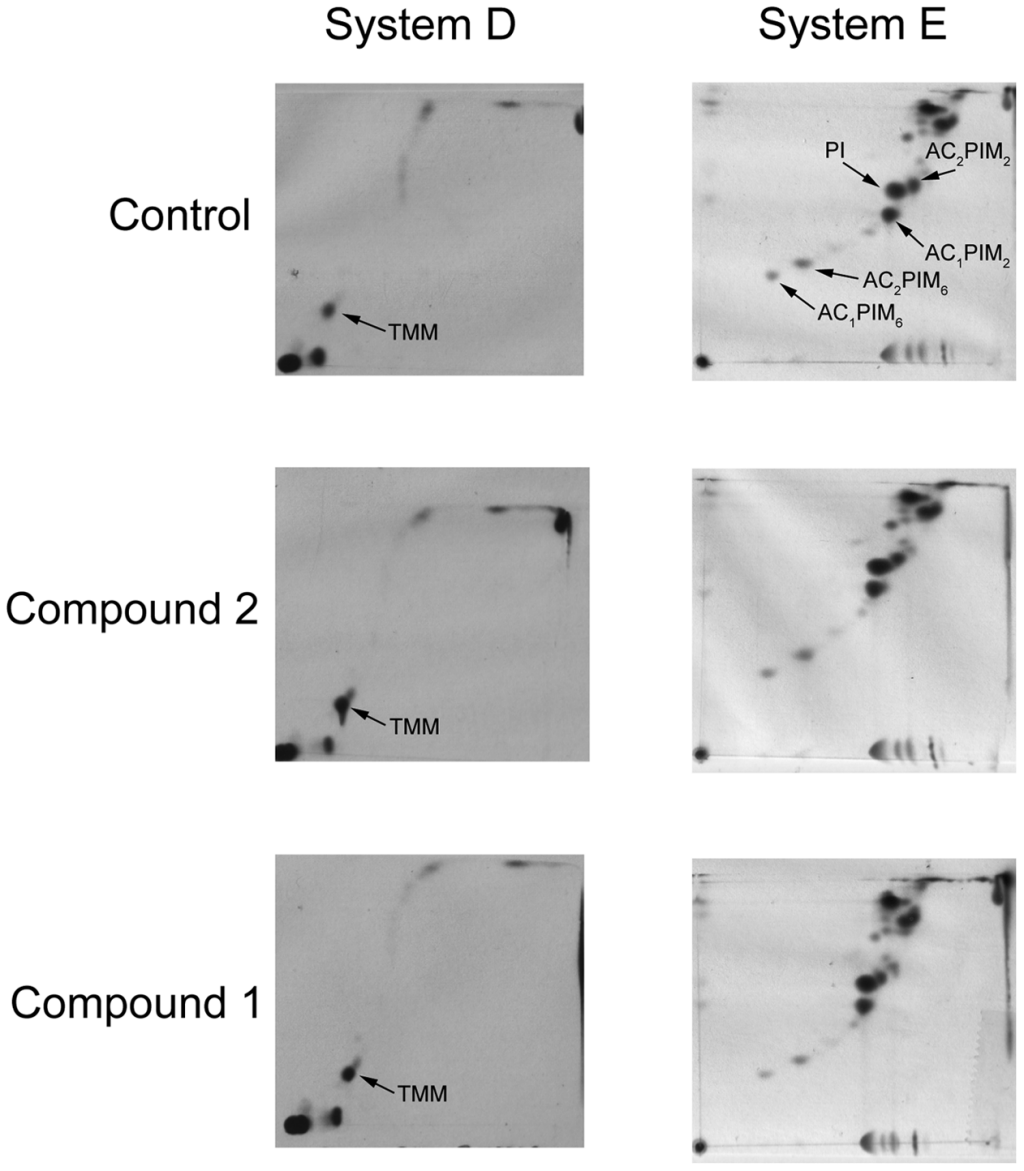
2D-TLC analysis of [^14^C]-labelled lipids from *M. bovis* BCG grown in the presence of MmpL3 inhibitors. Cultures were grown in the absence or presence of inhibitor (3× MIC) for 8 hours, and then and labelled using [^14^C]acetate for 8 hours. Chloroform-methanol extracts (polar lipids) were separated using (i) System D: chloroform:methanol:water (100∶14∶0.8) in direction 1 and chloroform:acetone:methanol:water (50∶60∶2.5∶3) in direction 2 with the position of TMM is indicated by the solid arrows; (ii) System E: chloroform:methanol:water (60∶30∶6) in direction 1 and chloroform:acetic acid:methanol:water (40∶25∶3∶6) in direction 2 with the position of phosphatidylinositol (PI) and phosphatidylinositol mannosides (PIMs) indicated. Lipids were visualized by 48 h exposure on X-ray films by autoradiography (Kodak Biomax MR film).

Recently, spontaneous mutants resistant to BM212, SQ109, adamantyl ureas and benzimidazole C215, compounds with anti-TB activities, were shown to contain mutations mapping to *mmpL3* [15],[16],[17]. Bacilli exposed to SQ109 and adamantyl ureas were shown to accumulate TMM suggesting that MmpL3 was involved in the export of TMM [14], [15]. To address the putative role of MmpL3 in TMM export, we assessed the effects of increasing concentrations of **1** and **2** on lipid metabolism in *M. bovis* BCG. Cultures of *M. bovis* BCG were grown in broth in the presence or absence of inhibitor for 8 hours, then labelled with [^14^C]-acetate for a further 8 hours and then subjected to an organic extraction procedure. In contrast to the cultures grown in the absence of inhibitor, [^14^C]-labelled cultures grown either in the presence of **1** and **2** showed an accumulation of TMM (Figure 3). To rule out non-specific effects, other classes of lipids, in particular phospholipids characteristic of mycobacteria were shown to be unaffected upon exposure to **1** and **2** (Figure 3). The accumulation of TMM by **1** and **2** suggests that biochemically they target MmpL3 and lead to a characteristic phenotypic profile as observed for other inhibitor families of MmpL3.

### Lead optimization summary, new leads profile

**Figure 4:**
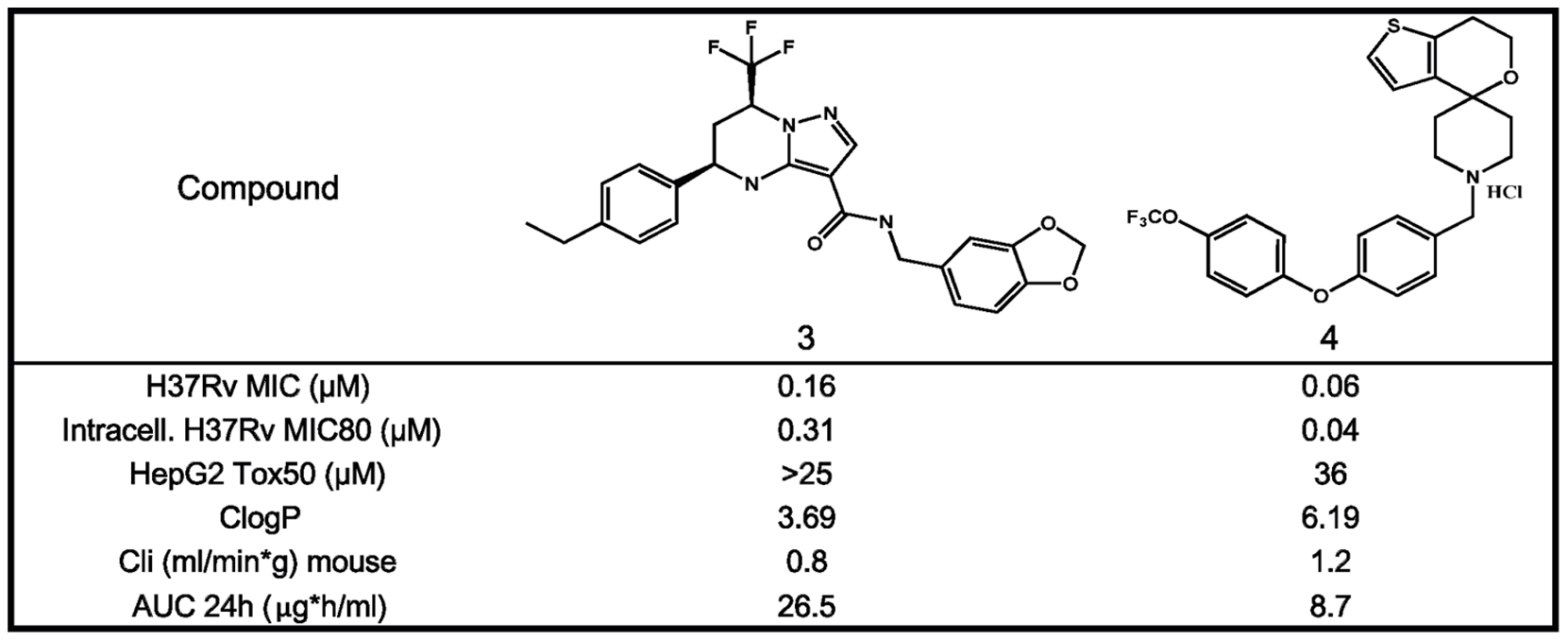
Profile of lead compounds 3 and 4. Structure, *in vitro* antimycobacterial activity against *M. tuberculosis* H37Rv, intracellular activity against *M. tuberculosis* H37Rv (RAW264.7 macrophages), cytotoxicity in HepG2 cells, ClogP, clearance in mouse microsomes, % PPB and areas under the curve versus time (AUC) after oral administration (po 50 mg/kg) of compounds **3** and **4**.

**Figure 5:**
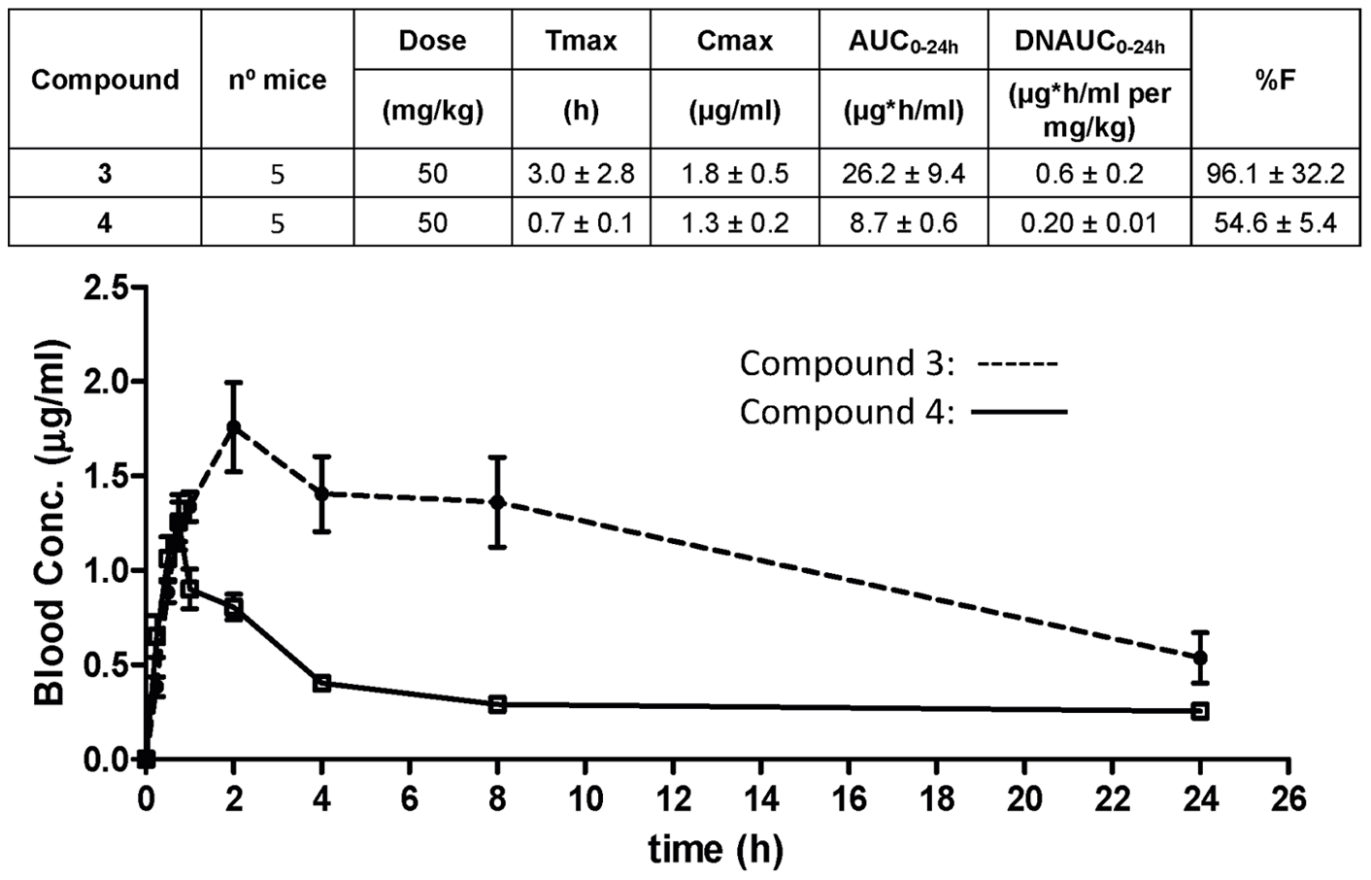
Whole blood pharmacokinetic profile and main parameters of compounds 3 and 4. Compounds were given orally at 50 mg/Kg suspension in 1% aqueous methylcellulose. Main pharmacokinetic parameters were established after non-compartimental analysis. AUC: Area Under the Curve; Cmax: Maximum concentration observed in whole blood; %F: percentage bioavailability.

Despite the interesting *in vitro* anti-tubercular profile stated in Table 1, the initial hits from both series were affected by a number of compound development liabilities (*in vitro* Drug metabolism and pharmacokinetics DMPK and physicochemical properties) that make difficult their progression to the available *in vivo* acute murine TB efficacy model. Further medicinal chemistry efforts (details to be reported elsewhere) were hence dedicated to the identification of optimized compounds that were able to retain or even improve the anti-tubercular profile while simultaneously meets good oral pharmacokinetic profile. These efforts led to the identification of **3** and **4** as new optimized lead representative structures for both chemical series (Figure 4). Both compounds retained or even improved the previous leads *in vitro* potency values against H37Rv and were able to reach acceptable exposure levels in blood after a single oral administration at 50 mg/kg (Figure 5). Cross resistance experiments with previously isolated mutants proved that the observed TB activity for both new lead compounds was target related (Table 3). Therefore, compounds **3** and **4**, despite the fact that some properties still remained to be optimized (high ClogP), fulfilled our minimum requirements in terms of *in vitro* potency (MIC <0.5 µM) and PK properties (AUC after oral administration 50 mg/kg >1 µg*h/mL) to be progressed to *in vivo* proof of concept efficacy studies in an acute TB murine infection model.

### *In vivo* efficacy of 3 and 4 in acute TB murine model

**Figure 6:**
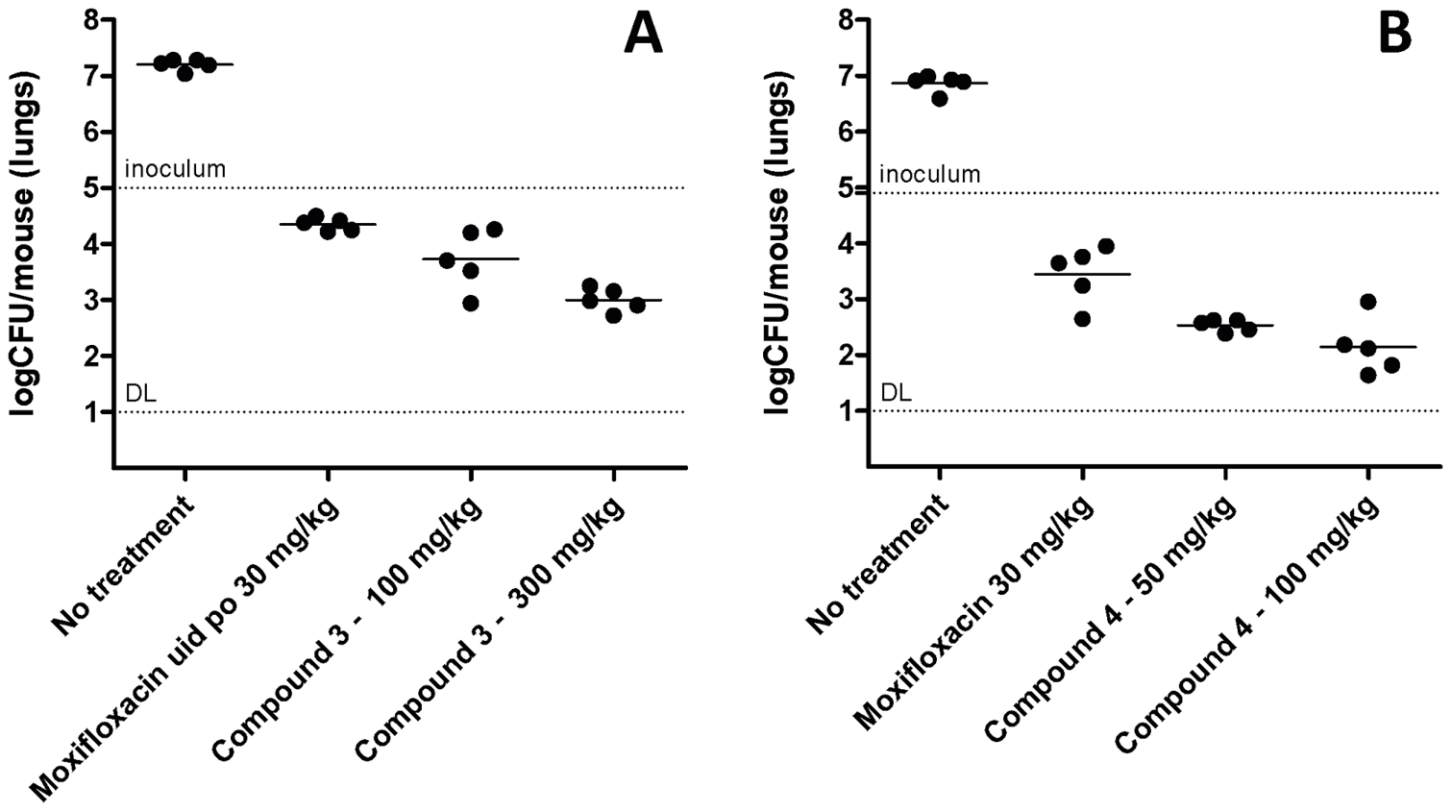
Therapeutic efficacy of Compound 3 (A) and Compound 4 (B) against H37Rv in vivo. B6 mice were infected by intratracheal instillation with 10^5^ CFU H37Rv per mouse. The mice were treated orally once a day from day 1 to day 8 and sacrificed on day 9. Every point represents data from lungs of one mouse. Moxifloxacin (30 mg/kg) was used as a quality control of the assay. DL: Limit of detection.

Efficacy studies were performed as previously described [20]. Under these conditions, both compounds showed a clear bactericidal effect, demonstrating a more than 1.5 log CFU reduction with respect to bacterial burdens at start of treatment (Figure 6). Compound **3** showed a reduction of 1.5 log and 2.2 log units in lung bacterial load when administered at recommended tolerable doses of 100 and 300 mg/kg respectively. In the case of compound **4**, it showed a reduction of 2.3 and 2.7 log units in the lungs of infected mice at recommended tolerable doses of 50 and 100 mg/kg respectively.

## Discussion

The *M. tuberculosis* genome encodes 13 members of the MmpL (*m*ycobacterial *m*embrane *p*rotein, *l*arge) family [21]. Our previous studies have shown that *mmpL3* specifically plays a key role in mycolic acid transport and ‘gene depletion’ in a conditional mutant resulted in the intracellular accumulation of TMM [19]. These studies were supported by *mmpL3*-inhibitor related studies [14],[15] which resulted in chemical phenotype identical to our genetic phenotype observed in a *M. smegmatis* conditional mutant [19]. The identification of *mmpL3* as an essential membrane protein-encoding gene involved in mycolate transport opens up a new avenues for targeting this essential and under-exploited mycobacterial pathway for developing new anti-TB drugs. In this present study, following our initial hit-to-lead identification of two novel series of anti-TB compounds, THPP and Spiro, and through the isolation of spontaneous resistant *M. tuberculosis* mutants followed by subsequent whole genome sequencing identified a number of mutations in *mmpL3* (Rv0206c) suggesting a likely mode of resistance to the THPP and Spiro agents. In all cases, the observed mutation is a single base change in *mmpL3* conferring a predicted amino acid alteration in the protein sequence. The majority of the mutations identified in *mmpL3* (57 out of 67) map to the first six transmembrane segments of the predicted MmpL3 topology and none of them coincide with the mutations previously isolated for other reported MmpL3 inhibitors (Table S1). The fact that most of the mutated amino acids are located within predicted transmembrane segments is not surprising given the hydrophobicity of the THPP and Spiro series of compounds. However, if these amino acids do interact directly with THPP and Spiro they might well inhibit MmpL3's ability to act as a TMM transporter. This is supported by our observations of THPP and Spiro-treatment of *M. bovis* BCG leading to an accumulation of TMM. As initial step to support MmpL3 as the target of the two compounds and to establish possible cross-resistance, resistant mutants were tested against each of the respective compounds. The results in this study concluded that both series did not possess significant cross-resistance against each other, pointing potentially to a different mechanism of resistance/action.

In order to characterise the *in vitro* bactericidal behaviour of leads **1** and **2**, the anti-tubercular kill rate of both molecules was monitored over a period of 7 days. Both compounds were able to reduce cfu counts at rates superior to Linezolid and comparable to Moxifloxacin and their behaviour could be clearly defined as bactericidal. Reassuringly, both compounds showed stable MIC values against a panel of susceptible, mono-resistant, MDR and XDR strains. While this panel was not intended to be representative of the genetic diversity within the *M. tuberculosis* complex, it gave an early indication of the therapeutic potential of this mode of action to tackle TB.

While a number of putative MmpL3 inhibitors with potent anti-tubercular *in vitro* activity have been previously reported [15],[16],[17], we have been able to identify and optimize two novel families of inhibitors (THPP and Spiro's) with lead representatives also capable of producing a significant efficacious response in a murine model of acute TB infection after oral administration. According to this model, the compounds are competitive when compared in terms of dosing to Moxifloxacin, an anti-tubercular drug candidate currently in Phase III clinical trials [20].

Despite the early optimisation of potency and microsomal clearance, compound **4** was found to still suffer from a high clogP value, with the consequent potential liabilities for further development [22]. In the case of compound **3**, potential issues associated were the mechanisms of potential drug-drug interaction and toxicity of benzodioxolane containing compounds which have been thoroughly reported elsewhere [23],[24] and highlights the need to develop new inhibitors where the presence of this derivative is avoided. Current synthetic efforts are focused on the optimization of these parameters and will be reported in due course.

## Materials and Methods

### General aspects and ethics Statement

All the experiments are approved by the Diseases of the Developing World, GlaxoSmithkline Ethical Committee. The animal research complied with Spanish and European Union legislation on Animal Research and GlaxoSmithKline policy on the Care and Use of Animals. Specific Pathogen-free 6–8-week-old female C57BL/6j mice (18–20 g) are obtained from Harlan (Harlan Interfauna Iberica, Spain). The experiments were performed at AAALAC-accredited GlaxoSmithKline Laboratory Animal Science animal facilities in Tres Cantos (Madrid, Spain). The mice were kept in air-conditioned facilities with fifteen air changes per hour. Room temperature and relative humidity were 22±3°C and 40–70%, respectively. The mice were accommodated in groups of up to five individuals in Tecniplast® type IV cages with autoclaved dust free corncob bedding (Panlab, Barcelona, Spain). The mice were maintained under a twelve hours light/dark period. Autoclaved tap water and irradiated pelleted diet were provided ad libitum. The compounds used in these *in vivo* studies were prepared as suspensions in 1% Methyl Cellulose. The antitubercular standards used in the efficacy study were: Moxifloxacin (Sequoia Research Products Ltd) prepared as solution in 20% Captisol(R)/water).

### MIC determination against mycobacteria

The measurement of the Minimum Inhibitory Concentration (MIC) against *M. tuberculosis* strains for each tested compound was performed in 96-well flat-bottom, polystyrene microtiter plates in a final volume of 100 μl. Ten two-fold drug dilutions in neat DMSO starting at 50 mM were performed. Drug solutions were added to Middlebrook 7H9 medium (Difco) and Isoniazid (INH) (Sigma Aldrich) was used as a positive control with two-fold dilutions of INH starting at 160 μg/ml. The inoculum was standardized to approximately 1×10^7^ cfu/ml and diluted 1 in 100 in Middlebrook 7H9 broth (Difco). This inoculum (100 μl) was added to the entire plate but G-12 and H-12 wells were used as blank controls. All plates were placed in a sealed box to prevent drying out of the peripheral wells and incubated at 37°C without shaking for six days. A Resazurin solution was prepared by dissolving one tablet of resazurin (Resazurin Tablets for Milk Testing; Ref 330884Y' VWR International Ltd) in 30 ml of sterile PBS (phosphate buffered saline). Of this solution, 25 μl were added to each well. Fluorescence was measured (Spectramax M5 Molecular Devices, Excitation 530 nm, Emission 590 nm) after 48 hours to determine the MIC value.

### MIC against Clinical strains

The BACTEC MGIT 960 System (Becton Dickinson) was used to MIC determination in clinical isolates (Institute Carlos III and Hospital Val d'Hebron) following the manufacturer instructions.

### MIC intracellular determination

Confocal automated plate reader (PE Opera) was used to assess the intracellular growth and proliferation of GFP expressing *M. tuberculosis* H37Rv in Raw264.7 macrophages as well as the mortality of host cells. In this experiment macrophages were infected as a batch with the batch preparation of mycobacteria (MOI  = 1) for 2 hours and then treated with amikacin to remove extracellular bacilli. Infected cells were plated into 384 well micro-titer plates containing compounds and incubated for 5 days. After incubation cell-permeable Syto60 was added to each well for macrophage nuclei staining. Images of cells in each well (several image fields per well) were recorded on an automated Opera platform located in a BSL3 facility. The percent of infected cells containing GFP-expressing bacteria were used as primary readout. The decrease in % of GFP containing cells was reflecting the ability of compounds to reduce bacterial growth, proliferation and infectivity. The total number of macrophages, determined by quantification of Syto60 stained nuclei, was used as a second independent readout in assay. Ten point dose titrations will be used for pharmacological characterization of compounds. The decrease of bacteria load with simultaneous increase in number of surviving host macrophages determined the minimal inhibitory concentration and effective concentration of inhibitors. Positive controls wells were used with INH and Rifampin at MIC 100 concentration and negative control wells with 1% DMSO on every plate in a screening run. These controls allowed normalizing data to 100% of inhibition on the plate-based level thus avoiding variability of results during multiple days of screening.

### General antimicrobial activity assay

Whole-cell antimicrobial activity was determined by broth microdilution using the Clinical and Laboratory Standards Institute (CLSI) recommended procedure, Document M7-A7, “Methods for Dilution Susceptibility Tests for Bacteria that Grow Aerobically”. Some compounds have been evaluated against a panel of Gram-positive and Gram-negative organisms, including *Enterococcus faecium*, *Enterococcus faecalis*, *Haemophilus influenzae*, *Moraxella catarrhalis*, *Streptococcus pneumoniae*, *Escherichia coli* and *Streptococcus pyogenes*. The MIC was determined as the lowest concentration of compound producing a >80 % reduction in fluorescence observed.

### HepG2 cytotoxicity assay

Cytotoxicity assay was performed measuring the viability of immortalized human cell line culture (HepG2) after 48 h of incubation with a full curve concentration of the test substances in culture medium. For read out of viability of cells we used Resazurine (BDH®). Resazurin is used as an oxidation-reduction indicator that yields a colorimetric change and a fluorescent signal in response to metabolic activity. As cell grow, metabolic activity results in a chemical reduction of Resazurin indicated by a change from non-fluorescent blue to the reduced fluorescent pink form. The degree of Resazurin fluorescence is therefore, an indicator of the number of viable cells in the culture system. The cytotoxicity results were expressed as Tox50 values (IC50). This was done by calculating the concentration of test compounds that would result in a decrease in Resazurin reduction equivalent to 50% of the concurrent control values. The protocol we followed is described below briefly.

Cell line HepG2 (HB-8065) were cultured with fresh medium (Essential Minimum Eagle Medium, EMEM, supplemented with 5% fetal calf serum and 2 mM L-glutamine) the day before subculturing the plates. On the day of the assay, cells (10,000 cells/well) were seeded in a black 96-well collagen coated microplate with clear bottom, (Becton Dickinson®) except in column 11, that was dispensed only 100 μL of culture medium.

Stock solution from each test substances was prepared in 100% DMSO. Ten serial 1∶2 dilutions were prepared of each test compound and, finally, a 1∶200 dilution was made, in medium, to achieve a final concentration of 0.5% of DMSO. Resazurin tablets (Merck®) were dissolved in phosphate buffer saline at a concentration of 0.0042%.

After 24 h of incubation of the cells (37°C, 5% CO_2_, 95% relative humidity), a volume of 150 μL of culture medium containing the appropriate test concentrations of the compounds dilutions were added to cells in two replicates. In column 12, only 150 μL of 0.5% DMSO was added (blank control). Then, cells were exposed to test compounds for 48 h. After that, medium was removed and Resazurin solution was added to each well and incubated for further 1.5 h. Fluorescence was measured at an excitation wavelength of 515 nm and an emission wavelength of 590 nm in a Microplate reader 1420 Multilabel HTS counter, Victor 2, (Wallac®) The fluorescence value of each well is corrected by subtracting the background value (average of column 11) from the absolute value. The percentages of inhibition are calculated relatively to the DMSO control wells (average of column 12). For each compound, the average value of the duplicate samples is calculated. Data were processed using an Excel spreadsheet and GraphPad Software analysis. Tox50 values were calculated from the Sigmoidal dose-response (variable slope) curves by nonlinear regression analysis.

### Microsomal fraction stability experimental procedure

Pooled mouse, rat, dog and human liver microsomes were purchased from Xenotech. Microsomes (final protein concentration 0.5 mg/ml, MgCl_2_ (final concentration 5 mM) and test compound (final substrate concentration 0.5 µM; final DMSO concentration 0.5 %) in 0.1 M phosphate buffer pH 7.4 were pre-incubated at 37°C prior to the addition of NADPH (final concentration 1 mM) to initiate the reaction. The final incubation volume was 600 µl. Control incubation was included for each compound tested where 0.1 M phosphate buffer pH 7.4 was added instead of NADPH (minus NADPH). One control compound was included with each species. All incubations were performed singularly for each test compound. Each compound was incubated for 30 minutes and samples (90 µl) of incubate were taken at 0, 5, 10, 20 and 30 minutes. The control (minus NADPH) was sampled at 0 and 30 minutes only. The reactions were stopped by the addition of sample to 200 µl of acetonitrile:methanol (3∶1) containing an internal standard. The terminated samples were centrifuged at 3700 rpm for 15 minutes at 4°C to precipitate the protein. Quantitative analysis: following protein precipitation, the samples were analyzed using specific LC-MS/MS conditions. Data analysis: from a plot of ln peak area ratio (compound peak area/internal standard peak area) against time, the gradient of the line was determined. Subsequently, half-life and intrinsic clearance were calculated using the equations below:

Elimination rate constant (k) = (-gradient) Half life 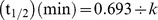


Intrinsic Clearance (CLint) (ml/min/g protein)  = 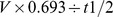 where V = Incubation volume ml/g microsomal protein.

### Killing kinetics assay

Bacteria were grown at 37°C in 7H9 broth ADC Tween to mid-exponenetial phase and then diluted in 10 ml fresh Middlebrook 7H9 to an 5×105 cfus/ml. Incubation was continued after the addition of compounds at 20X the MIC. At specified time points, aliquots of cultures were withdrawn, serially diluted in 7H9 broth Tween and plated on solid culture medium. Plates were then incubated at 37°C and CFU were counted after 3 to 4 weeks.

### Polar and apolar lipid extraction of [14C]-labelled inhibitor-treated cultures of *M. bovis* BCG

A 5 ml culture of *M. bovis* BCG was grown until mid-log (OD600 0.6), then inhibitor (1 or 2) added at 3× MIC and the culture grown for a further 8 hours at 37°C. At this point, the cells were labelled with 10 µCi ml-1 [1,2–14C]acetate (57 mCi mmol-1, GE Healthcare, Amersham Bioscience), and incubated at 37°C for a further 8 hours. The [14C]-labelled cells were harvested by centrifugation followed by washing with PBS. Polar and apolar lipids were then extracted according to the procedures described by Dobson et al. [25]. Briefly, freeze-dried M. bovis BCG cells were treated in 2 ml of methanolic saline (CH3OH:0.3% NaCl, 10∶1, v/v) and 2 ml of petroleum ether for 2 h [25]. The suspension was centrifuged and the upper layer containing apolar lipids was separated. An additional 2 ml of petroleum ether was added, mixed and centrifuged as described above. The two upper petroleum ether fractions were combined and dried. For polar lipids, 2.3 ml CHCl3/CH3OH/0.3% NaCl (9∶10∶3, v/v/v) was added to the lower aqueous phase and stirred for 4 h. The mixture was centrifuged, separated and the residue re-extracted twice with 0.75 ml of CHCl3/CH3OH/0.3% NaCl (5∶10∶4, v/v/v). Equal amounts of CHCl3 and 0.3% NaCl (1.3 ml of each) were added to the combined filtrates and stirred for 1 h. The mixture was centrifuged and the lower layer containing the polar lipids recovered, dried, and resuspended in 200 ml of CHCl3:CH3OH (2∶1, v/v), and incorporation of [14C] acetate quantified by liquid scintillation counting using 5% of the lipid fraction in 5 ml of EcoScint A (National Diagnostics). The polar lipid extracts were examined by two dimensional thin-layer chromatography (2D-TLC) on aluminum backed plates of silica gel 60 F254 (Merck 5554) by spotting equal counts of polar lipid extracts (50, 000 cpm), which were then developed using solvent systems D and E as described [25]. System D: chloroform:methanol:water (100∶14∶0.8) in direction 1 and chloroform:acetone:methanol:water (50∶60∶2.5∶3) in direction 2; and System E: chloroform:methanol:water (60∶30∶6) in direction 1 and chloroform:acetic acid:methanol:water (40∶25∶3∶6) in direction 2. Lipids were visualized by 48 h exposure on X-ray films by autoradiography (Kodak Biomax MR film).

### Pharmacokinetic studies

Experimental compounds were administered by oral gavage at 50 mg/kg single dose at a volume of 20 ml/kg to n = 5 mice. All mice received treatment in the fed state. Peripheral total blood was the compartment chosen for the establishment of compound concentrations: 25 μl of blood was taken from the lateral tail vein for each mouse at 15, 30 and 45 minutes, 1, 2, 4, 8 and 24 hours. LC-MS was used as the analytical method of choice for the establishment of compound concentration in blood with a sensitivity of LLQ  = 1–5 ng/ml in 25 µl blood. The non-compartmental data analysis (NCA) was performed with WinNonlin Phoenix 6.3 (Pharsight, Certara L.P) and supplementary analysis was performed with GraphPad Prism 5 (GraphPad Software, Inc).

### *In vivo* efficacy assessment

The experimental design has been previously described [20]. In brief, mice were intratracheally infected with 100.000 CFU/mouse (*M. tuberculosis* H37Rv strain). Products were administered for 8 consecutive days starting one day after infection. Lungs were harvested 24 hours after the last administration. All lung lobes were aseptically removed, homogenized and frozen. Homogenates were plated in 10% OADC-7H11 medium for 14 days at 37°C. Homogenates from compound **3** and **4** treated mice were incubated for 18 days at 37°C in plates supplemented with 0.4% (wt, vol) activated charcoal (Sigma Aldrich) to prevent the effect of product carryover.

## Supporting Information

Protocol S1 **Compounds synthesis.** This section provides a detailed description of the protocols, experimental procedures and specifics of how compounds **1–4** were obtained. It also includes information on materials used in the study and references.
(DOCX)

Protocol S2 **Growth and generation of** ***M. tuberculosis*** **spontaneous resistant mutants.** This section provides a detailed description of the protocols, experimental procedures and specifics of how mutants were obtained. It also includes sequencing methods.
(DOCX)

Protocol S3 **Determination of absolute configuration of compound 1 through Vibrational Circular Dichroic (VCD).**
(DOCX)

Table S1 **List of mutations with different MmpL3 inhibitors.** Comparison of mutations identified in mmpL3 with GSK compounds and previously isolated for other reported MmpL3 inhibitors.
(DOCX)
