# Supplementary material for: Tetrahydropyrazolo[1,5-a]Pyrimidine-3-Carboxamide and N-Benzyl-6′,7′-Dihydrospiro[Piperidine-4,4′-Thieno[3,2-c]Pyran] Analogues with Bactericidal Efficacy against Mycobacterium tuberculosis Targeting MmpL3
Source: PLoS One. 2013 Apr 17;8(4):e60933. doi: 10.1371/journal.pone.0060933 (PMC3629081; doi:10.1371/journal.pone.0060933)
Supplement: Protocol S1 — Compounds synthesis. This section provides a detailed description of the protocols, experimental procedures and specifics of how compounds 1–4 were obtained. It also includes information on materials used in the study and references. (DOCX) [file pone.0060933.s001.docx]

**Protocol S1 Compounds synthesis.**

**General information**

All commercially available reagents and solvents were used without further purification. When reactions were performed under microwave conditions, a Biotage Initiator was used. Automated flash chromatography was performed on a Biotage FlashMaster II system with peak detection at 254 nm. All products were obtained as amorphous solids and melting points were not measured. ^1^H NMR spectra were recorded at 400 MHz on a Bruker Ultrashield DPX 400 spectrometer. Chemical shifts (δ) are given in ppm relative to the solvent reference as an internal standard (d_6_-DMSO, δ = 2.50 ppm; CDCl_3_, δ = 7.27 ppm; CD_3_OD, δ = 3.31 ppm). Data are reported as follows: chemical shift (multiplicity (s for singlet, d for doublet, t for triplet, m for multiplet, br for broad), integration, coupling constant(s) in Hz). HPLC–MS analyses were conducted on an Agilent 1100 instrument equipped with a Sunfire C18 column (30 mm x 2.1 mm i.d., 3.5 mm packing diameter) at 40 ºC coupled with a Waters ZMD2000 mass spectrometer; the method of ionization was alternate-scan positive and negative electrospray. Semi-preparative chiral HPLC were conducted on an Agilent 1100 instrument equipped with a Chiralpack IC column (250 x 20 mm). Preparative chiral HPLC were conducted on a Varian SD-2 prep HPLC instrument equipped with a Chiralpak IC column ( 250 x 50mm i.d, 20 um). Compounds had purity of >95%, as determined by HPLC and 1H NMR analysis.

**(5R,7S)-5-(4-ethylphenyl)-N-(4-methoxybenzyl)-7-(trifluoromethyl)-4,5,6,7-tetrahydropyrazolo[1,5-a]pyrimidine-3-carboxamide (1)**. Cis-5-(4-ethylphenyl)-7-(trifluoromethyl)-4,5,6,7-tetrahydropyrazolo[1,5-a]pyrimidine-3-carboxylic acid [1] (618 mg, 1.82 mmol), HATU (831 mg, 2.18 mmol) and N,N-diisopropylethylamine (0.954 mL, 5.46 mmol) was dissolved in N,N-dimethylformamide (DMF) (15mL). The reaction mixture was stirred for 30 min at rt then 4-methoxybenzylamine (300 mg, 2.18 mmol) was added. The reaction was stirred at 60 ºC overnight. The reaction was checked by LCMS and the reaction was completed. The reaction mixture was concentrated under vacuum and partitioned between sodium carbonate (10%) 50 mL and DCM (50 mL) the organic layer was separated, dried over Na_2_SO4, filtered and concentrated under vacuum. The crude product was added to a silica gel column (30 g) and was eluted with (gradient 100% hexane to hexane/EtOAc 50/50), after collected the appropriated tubes the solvent was removed under vacuum to afford a pale yellow solid (780 mg, 1.70 mmol 93%) ^1^H NMR (400 MHz, CDCl_3_) δ ppm: 7.50 (s, 1H), 7.35 (d, 2H), 7.27-7.22 (m, 4H), 6.88 (d, 2H, J=8.1), 6.68 (bs, 1H), 4.87-4.81 (m, 1H), 4.53 (dd, 1H, J=2.5 and 8.6), 4.48-4.47 (m, 2H), 3.80 (s, 3H), 2.66 (q, 2H, J=7.6), 2.55-2.52 (m, 1H), 2.42-2.33 (m, 1H), 1.25 (t, 3H, J=7.6). [ES+ MS] m/z 459 (M+H)+. Racemic compound was subjected to semi-preparative high performance liquid chromatography (HPLC) to afford the optically pure enantiomer **1** $[{a]}_{D}^{25}$= ­43.0 (c = 0.44, EtOH). (5R,7S) >95% ee (shorter retention time) and corresponding enantiomer **1b** $[{a]}_{D}^{25}$= -49.2 (c = 0.30, EtOH) (5S, 7R), >95% ee (longer retention time). [Column: Chiralpack IC, 250 x 20 mm, temperature 25ºC, mobile phase: hexane/ethanol 90/10, Flow rate: 18 mL/minute, detection wavelength: 254 nm, and injection of 100 mg. The absolute configuration of the enantiomers **1** and **1b** was subsequently determined by ab initio vibrational circular dichroism (VCD) with a level of reliability > 99%. See additional supplementary material.

**(5R,7S)-5-(4-ethylphenyl)-7-(trifluoromethyl)-4,5,6,7-tetrahydropyrazolo[1,5-a]pyrimidine-3-carboxylic acid.** 30g of racemic carboxylic acid [1] was diluted with ethanol (400mL) and warmed in a sonicating bath at 40ºC for 1hr. To the hazy solution (contained white flocculent solid) was added heptane (400mL) and the mixture further sonicated at 40ºC for 1h. Ethanol (50mL) added and the hazy solution warmed to approx. 55ºC with stirring. The warm hazy solution was filtered (porosity 3 filter) before injection. The following conditions were then employed to process the clarified solution on the Varian SD-2 prep HPLC system; Column: Chiralpak IC, 250 x 50mm ID, 20um; Eluent: Heptane+0.1%v/v Acetic acid/Ethanol (90:10v/v); Flow: 125mL/min; Temp: 25ºC; Detector: 280nm, Range 5. Injection: 37x22mL & 17mL of 30.9mg/mL solution in Heptane/Ethanol (47:53v/v). (Injection pump set to flow at 46mL/min for 30sec which actually delivers 22 mL). Fractions which contained the desired first eluting enantiomer were combined (approx 10L) and evaporated on the 20L Buchi using a 10L flask until the majority of solvent had been removed, then the residue was diluted with ethanol (total vol 200mL, includes flask rinse) and the pale yellow solution transferred to a 1L flask. The solvent was removed by evaporation (70mbar/40ºC) on a lab Buchi and the resulting pale yellow 'oily' foam diluted with heptane (100mL). After re-concentration the residue was triturated with heptane (100mL) by agitation on a Buchi at 40ºC for 1hr and occasional scraping with a spatula. The resulting white suspension was allowed to stand at ambient temperature overnight, then filtered. The collected solids were washed with heptane (2x50mL), sucked dry on the filter for ca 2 h. and then further dried in vacuo at 45ºC for a total of 22.5 h. to furnish the first eluting enantiomer (5R,7S), as a white solid. 10 g isolated (36%w/w recovery). Chiral HPLC showed a purity of 99.1% and no evidence of the second eluting enantiomer $[{a]}_{D}^{25}$= = + 41.4 (c = 0.56, EtOH). Fractions rich in the second eluting enantiomer were combined (approx 20L) and evaporated on the 20L Buchi using a 10L flask until the majority of solvent had been removed, then the residue was diluted with ethanol (total vol 200mL, includes flask rinse) and the pale yellow solution transferred to a 1L flask. The solvent was removed by evaporation (70mbar/40ºC) on lab Buchi and the resulting yellow oil diluted with heptane (100mL). After re-concentration the residue was triturated with heptane (100mL) by agitation on a buchi at 40ºC for 1hr and scraping with a spatula. The resulting white suspension was allowed to stand at ambient temperature overnight. The solids were collected by filtration, then washed with heptane (2x50mL), sucked dry for ca 1 h. and further dried in vacuo at 45ºC for a total of 65 h. to afford the second eluting enantiomer **(5S,7R)**, as a white solid 8.2 g isolated (30%w/w recovery). Chiral HPLC showed a purity of 98.3%.$[{a]}_{D}^{25}$= - 45.2 (c = 0.76, EtOH).

**(5R,7S)-N-(benzo[d][1,3]dioxol-5-ylmethyl)-5-(4-ethylphenyl)-7-(trifluoromethyl)-4,5,6,7-tetrahydropyrazolo[1,5-a]pyrimidine-3-carboxamide (3).** To a solution of (5R,7S)-5-(4-ethylphenyl)-7-(trifluoromethyl)-4,5,6,7-tetrahydropyrazolo[1,5-a]pyrimidine-3-carboxylic acid (4.5 g, 13.26 mmol) in N,N-Dimethylformamide (DMF) (25 mL) was added HATU (6.05 g, 15.91 mmol) and N,N-diisopropylethylamine (6.95 mL, 39.80 mmol) , the mixture was stirred at room temperature for 30 min then piperonylamine (2.481 mL, 19.89 mmol) was added and the mixture was stirred at 50ºC overnight. After cooling to room temperature, reaction mixture was diluted with tert-butyl methyl ether (50 mL) and washed with sat. NH_4_Cl (25 mL) and sat. NaCl (25 mL). When it was washed with NaCl a solid appears and it was necessary to dilute with more tert-butyl methyl ether (100 mL). The organic layers were dried over MgSO_4_, filtered and evaporated. The crude was purified by grinding with a mixture of tert-butyl methyl ether (15 mL) and hexane (30 mL), but solid filtrate was pale brown and it was repurified solving solid with 5 mL of Dichloromethane and precipitating with Hexane (30 mL). Solid was filtered and dried in a vacuum drying overnight at 40ºC to obtain a white solid. (4.1 g, 862 mmol, 65.4%). ^1^H NMR (400 MHz, CDCl_3_) δ ppm: 7.51 (s, 1H), 7.35 (d, 2H, J=8.1), 7.23 (d, 2H, J=8.1), 6.82 (s, 1H), 6.77 (s, 2H), 6.67 (bs, 1H), 5.94 (s, 2H), 5.82-5.79 (m, 1H), 4.88-4.80 (m, 1H), 4.55-4.52 (m, 1H), 4.45-4.43 (m, 2H), 2.66 (q, 2H, J=7.6), 2.55-2.50 (m, 1H), 2.42-2.33 (m, 1H), 1.25 (t, 3H, J=7.6). [ES+ MS] m/z 473 (M+H)+. $[{a]}_{D}^{25}$= + 43.7 (c = 0.6, EtOH).

**1-(2,3-Dihydro-1,4-benzodioxin-6-ylmethyl)-6',7'-dihydrospiro[piperidine-4,4'-thieno[3,2-c]pyran] (2).** A mixture of 6',7'-dihydrospiro[piperidine-4,4'-thieno[3,2-c]pyran] [2] (280 mg, 1.30 mmol), 1,4-benzodioxan-6-carboxaldehyde (329 mg, 2.00 mmol), polymer supported cyanoborohydride (885 mg, 2.20-4.00 mmol), acetic acid (0.306 mL, 5.40 mmol) and dichloromethane (26 mL) was heated at 100 ºC for 45 min under microwave irradiation. Solids were filtered off, volatiles removed under vacuum and the residue was dissolved in MeOH and loaded onto a 20 g SCX ion exchange cartridge. By-products were eluted with MeOH and the product was eluted with a 7 M methanolic ammonia solution. Concentration under vacuum afforded a yellowish oil that was purified using a silica gel cartridge (Analogix, 12 g) and eluting with a mixture of methanol/dichloromethane (gradient 0-4%). Collected fractions were evaporated under reduced pressure to obtain the title compound 1 (323 mg, 0.900 mmol, 67 % yield) as a colourless oil. ^1^H NMR (400 MHZ, d_6_-DMSO): δ ppm: 7.26 (d, 1H, J=5.3), 6.94 (d, 1H, J=5.3), 6.80-6.73 (m, 3H), 4.21 (s, 4H), 3.83 (t, 2H, J=5.3), 3.36 (s, 2H), 2.73 (t, 2H, J=5.3), 2.61-2.54 (m, 2H), 2.30-2.21 (m, 2H), 1.89-1.80 (m, 2H), 1.75-1.67 (m, 2H). [ES+ MS] m/z 358 (M+H)+.

**1-(4-(4-(Trifluoromethoxy)phenoxy)benzyl)-6',7'-dihydrospiro[piperidine-4,4'-thieno[3,2-c]pyran], hydrochloride (4).** A mixture of 6',7'-dihydrospiro[piperidine-4,4'-thieno[3,2-c]pyran] [**2**] (368 mg, 1.75 mmol), 4-(4-(trifluoromethoxy)phenoxy)benzaldehyde (496.4 mg, 1.75 mmol) [3], polymer supported cyanoborohydride (1.16 g, 2.90-5.20 mmol), acetic acid (0.403 mL, 7.00 mmol) and dichloromethane (20 mL) was heated at 100 ºC for 2 h under microwave irradiation. The reaction mixture was filtered and washed with 10% Na_2_CO3 aqueous solution. The organic layer was dried over MgSO_4_, filtered and concentrated. The residue was purified by preparative HPLC using an XBRIDGE 30 x 150 mm column and eluting with a mixture of MeCN/10mM aqueous NH_4_HCO_3_ (gradient 70-100%) Collected fractions were evaporated under reduced pressure to obtain the desired compound as a free base (437 mg, 0.920 mmol). The compound was dissolved in 1 mL DCM and treated with HCl (3M in MeOH, 537 μl). Evaporation afforded the title compound 2 (460.9 mg, 0.900 mmol, 51 % yield) as a white solid after evaporation of volatiles. ^1^H NMR (400 MHZ, CD_3_OD): δ ppm: 7.54-7.52 (m, 2H), 7.32-7.30 (m, 2H), 7.22 (d, 2H, J=5.3), 7.12-7.08 (m, 4H), 6.84 (d, 2H, J=5.3), 4.16 (br s, 2H), 3.96 (t, 2H, J=5.3), 3.28-3.06 (m, 4H), 2.84 (t, 2H, J=5.3), 2.22-2.11 (m, 2H), 2.08-1.99 (m, 2H). [ES+ MS] m/z 476 (M+H)+.

References

1. Yoshida M, Mori A, Inaba A, Oka M, Makino H, et al. (2010) Synthesis and structure-activity relationship of tetrahydropyrazolopyrimidine derivatives-A novel structural class of potent calcium-sensing receptor antagonists. Bioorg Med Chem 18:8501-8511.

2. Shao L, Campbell JE, Hewitt MC, Campbell U, Hanania, TG (2011) Multicyclic compounds and methods of use thereof. WO 2011/069063 A2.

3. Yeates CL, Batchelor JF, Capon EC, Cheesman NJ, Fry M, et al. (2008) Synthesis and structure-activity relationships of 4-pyridones as potential antimalarials. J Med. Chem. 51:2845-52.
