# Supplementary material for: Tetrahydropyrazolo[1,5-a]Pyrimidine-3-Carboxamide and N-Benzyl-6′,7′-Dihydrospiro[Piperidine-4,4′-Thieno[3,2-c]Pyran] Analogues with Bactericidal Efficacy against Mycobacterium tuberculosis Targeting MmpL3
Source: PLoS One. 2013 Apr 17;8(4):e60933. doi: 10.1371/journal.pone.0060933 (PMC3629081; doi:10.1371/journal.pone.0060933)
Supplement: Protocol S2 — Growth and generation of M. tuberculosis spontaneous resistant mutants. This section provides a detailed description of the protocols, experimental procedures and specifics of how mutants were obtained. It also includes sequencing methods. (DOCX) [file pone.0060933.s002.docx]

**Protocol S2 Growth and generation of *M. tuberculosis* spontaneous resistant mutants**

*M. tuberculosis* resistant mutants were selected by plating 10^8^ mid-log (OD_600nm_, 0.8-1.0) cells on solid media containing 10x MIC of each compound. Resistant colonies were subsequently restricted in solid media in order to reisolate the colonies. A colony of each mutant was inoculated in liquid media with and without inhibitor and the MICs was determined to confirm that the resistance was genotypic and no phenotypic.. Following the validation of the mutants, 3 resistant mutants to each compounds were selected Purified genomic DNA from these selected spontaneous mutant, three each from 1 and **2**, and the wild-type strain (total of seven samples) were prepared for sequencing using the Beckman SPRIworks Fragment Library System I with adapters from the Illumina Multiplexing Sample Preparation Oligonucleotide kit. DNA (3 μg) was fragmented with a Bioruptor instrument (Diagenode, Lie, Belgium) using 100 μl volume and 30 cycles. The fragments were end-repaired, Illumina adapters were ligated and size selected (300-600 bp) using SPRIworks. The size-selected fragments were amplified (18 cycles using Phusion DNA Polymerase) and DNA was purified with Agencourt AMPure XP beads (Beckman Coulter Genomics, High Wycombe, UK). The median fragment size of the final libraries was 420-575 bp (assessed by a BioAnalyzer High Sensitivity LabChip, Agilent). Libraries were quantified with a Quant-iT PicoGreen dsDNA kit (Life Technologies), diluted to 10 nM, pooled and sequenced at the University of Warwick on an Illumina Genome Analyser IIx. Reads were aligned to the M. tuberculosis H37Rv reference genome sequence (Genbank accession: NC_000962.2) using the BWA aligner (version 0.6.1-r604) [pmid:19451168] using the aln/sampe pipeline with default settings, other than allowing for insert sizes up to 1000 bases in length. Putative variants were then detected using samtools/bcftools 0.1.18. Aligned BAM files were supplied as input to the samtools mpileup command using the command-line modifiers "-C50 -D -S -E" and otherwise default settings [pmid:19505943]. Low-quality variants were initially filtered to exclude those with a phred-scaled variant quality score of less than 60 and a strand bias score (Fisher's exact test) of greater than or equal to 0.005. The putative effect of SNPs on coding sequences was determined using snpEff 2.0.5d [pmid:22728672]. Following the identification of SNPs within the 6 whole genome sequenced mutants, mutations consistently mapped to Rv0206c (*mmpL3*) across all six strains. Subsequent targeted sequencing of *mmpL3* was performed on a further panel of 61 spontaneous *M. tuberculosis* resistant mutants to 1 and 2.

The *mmpL3* gene was amplified by PCR to generate a 3kb product. The sequences of the primers were as follows: 5'- AGGACATCGCTGGTGTTTTC -3' and 5'- CGGTCGCTTTCTTCAACAAT -3'. The following thermocycler parameters were used: an initial denaturation step of 5 min at 95ºC, followed by 35 cycles of 30 s at 95ºC, 30 s at 62ºC and 4 min at 72ºC and ending with a final elongation step of 10 min at 72ºC. A total of 18 Sanger sequencing primers were designed using Primer 3 to generate overlapping sequence reads across the 3kb sequence. Sequence reactions were conducted as follows. 50-80ng of PCR product was cycle sequenced on an MJ research thermocycler for 30 cycles according to standard ABI protocols using 0.25x BigDye® from the Terminator v3.1 Cycle Sequencing Kit . Reaction products were purified using the Agencourt cleanseq reagent and Beckman FX robotics. Samples were resuspended in 0.05mM EDTA and run on an Applied Biosystems 3730xl DNA Analyzer according to standard protocols. The raw data was analysed firstly using Sequence analysis v5.2 to generate sequence traces, followed by Seqscape v2.6 to detect the presence of mutations. The reference sequence used was NC_000962.2. Mutations found were translated to find potential amino acid substitutions using also the Seqscape v2.6 package.
