# Supplementary material for: Tetrahydropyrazolo[1,5-a]Pyrimidine-3-Carboxamide and N-Benzyl-6′,7′-Dihydrospiro[Piperidine-4,4′-Thieno[3,2-c]Pyran] Analogues with Bactericidal Efficacy against Mycobacterium tuberculosis Targeting MmpL3
Source: PLoS One. 2013 Apr 17;8(4):e60933. doi: 10.1371/journal.pone.0060933 (PMC3629081; doi:10.1371/journal.pone.0060933)
Supplement: Protocol S3 — Determination of absolute configuration of compound 1 through Vibrational Circular Dichroic (VCD). (DOCX) [file pone.0060933.s003.docx]

**Protocol S3: Vibrational Circular Dichroic Analysis of Enantiomers 1 and 1b**

The absolute configurations of **1** and **1b** were assigned using vibrational circular dichroism (VCD).

*Experimental:*

VCD spectra were acquired using a BioTools Dual-PEM ChiralIR^TM^ FT-VCD spectrometer operating at 4 cm^-1^ resolution, with modulators calibrated at 1400 cm^-1^ and retardations set at PEM1 = 0.250 λ, PEM2 = 0.260 λ. A total of 18720 scans were accumulated for each VCD measurement. Spectra were acquired using an International Crystal Laboratories sealed transmission cell with BaF_2_ windows and 100 micron pathlength. Samples were dissolved in CDCl_3_ at 0.2-M concentration. The baseline artifact inherent in experimental VCD spectra was removed by the half-difference correction method: VCD_corr’d_ (**1**) = [(VCD**_1_** – VCD**_1b_**)/2]; VCD_corr’d_ (**1b**) = [(VCD**_1b_** – VCD**_1_**)/2].

*Computational Analysis:*

The VCD analysis was performed using a model with (5S,7R) absolute configuration. The conformational set was identified using a molecular mechanics conformational search. Spectra were calculated for each conformation using the B3LYP/DGDZVP computational method. VCD and IR spectra were synthesized using Gibbs free energies and Boltzmann statistics. The calculated line spectra were fitted with Lorentzian band shapes using an 8 cm^-1^ resolution factor (hwhh) and a uniform scaling factor of 0.975. The level of confidence in the VCD assignments was estimated using CompareVOA^TM^ (BioTools, Inc.; Jupiter, Fla. USA), an algorithm that uses overlap integrals to quantify the agreement between calculated and observed VCD spectra.

*Data Analysis:*

The VCD and IR spectra observed for **1** and **1b** are compared with calculated spectra in Figures 1 and 2, respectively. In the top panel of Figure 1, the calculated VCD spectrum is the mirror image of the VCD spectrum of **1**, while in the top panel of Figure 2, the calculated spectrum is coincident with the VCD spectrum of **1b**. These data are consistent with **1** being the mirror image stereo isomer of the model and **1b** being the same stereo isomer as the model, leading to the assignment of **1**as the (5R,7S) enantiomer and **1b** as the (5S,7R) enantiomer. The confidence limit for these assignments was estimated to be >99%.

The calculated IR spectrum is in good qualitative agreement with experimental, indicating adequate coverage of conformational space. [Note: Misalignment of the CF_3_ symmetric (obs’d 1275 cm^-1^; calc’d 1235 cm^-1^) and antisymmetric (obs’d 1155 cm^-1^; calc’d 1120 cm^-1^) stretching modes is due to limitations/errors in the computation method used for this analysis. The signs of CF_3_ bands in the VCD spectra, however, are consistent with overall VCD comparisons (CF_3_ bands oppositely signed for **1** versus model comparison, same signs for **1b** versus model comparison.]

Figure S-1: VCD spectra of **1** and the (5S,7R) model (top); IR spectra of **1** and the 5S,7R) model (bottom).

Figure S-2: VCD spectra of **1b** and the (5S,7R) model (top); IR spectra of **1b** and the 5S,7R) model (bottom).
