# Supplementary material for: Tetrahydropyrazolo[1,5-a]Pyrimidine-3-Carboxamide and N-Benzyl-6′,7′-Dihydrospiro[Piperidine-4,4′-Thieno[3,2-c]Pyran] Analogues with Bactericidal Efficacy against Mycobacterium tuberculosis Targeting MmpL3
Source: PLoS One. 2013 Apr 17;8(4):e60933. doi: 10.1371/journal.pone.0060933 (PMC3629081; doi:10.1371/journal.pone.0060933)
Supplement: Table S1 — List of mutations with different MmpL3 inhibitors. Comparison of mutations identified in mmpL3 with GSK compounds and previously isolated for other reported MmpL3 inhibitors. (DOCX) [file pone.0060933.s004.docx]

**Table S1 List of mutations with different MmpL3 inhibitors.**

| **Transmembrane Domains** | | **Compounds used for mutant selection** | | | | | | |
| --- | --- | --- | --- | --- | --- | --- | --- | --- |
| **Nucleotide** | **Number** | **DA5  (Tahlan *et al*., 2012)** | **DA8  (Tahlan *et al*., 2012)** | **AU1235 (Grzegorzewicz *et al*., 2012)** | **BM212  (La Rosa *et al*., 2012)** | **Smeg^GSK2R^** | **GSK2** | **GSK1** |
| 14 – 34 | **1** |  |  |  |  |  |  |  |
|  |  |  | Q40R |  |  |  |  |  |
| 186 – 206 | **2** |  |  |  |  |  |  |  |
| 210 – 230 | **3** |  |  |  | L215S |  |  |  |
| 236 – 256 | **4** |  |  | G253E |  |  | Y252C | A249P |
|  |  |  |  |  |  |  | F255V |  |
|  |  |  |  |  |  |  | F255L |  |
| 287 – 307 | **5** |  |  |  |  | I297F | I292T |  |
|  |  |  |  |  |  |  | I292S |  |
| 563 – 583 | **8** |  | L567P |  |  |  |  |  |
|  |  |  |  |  |  |  | S591I |  |
| 617 – 637 | **10** |  |  |  |  |  |  |  |
|  |  |  |  |  |  |  |  | F644C |
| 673 – 693 | **11** |  |  |  |  |  |  | A677V |
| 699 – 719 | **12** | A700T |  |  |  |  |  | V713M |
